# Diagnosis of Choroidal Disease With Deep Learning-Based Image Enhancement and Volumetric Quantification of Optical Coherence Tomography

**DOI:** 10.1167/tvst.11.1.22

**Published:** 2022-01-14

**Authors:** Kazuichi Maruyama, Song Mei, Hirokazu Sakaguchi, Chikako Hara, Atsuya Miki, Zaixing Mao, Ryo Kawasaki, Zhenguo Wang, Susumu Sakimoto, Noriyasu Hashida, Andrew J. Quantock, Kinpui Chan, Kohji Nishida

**Affiliations:** 1 Department of Vision Informatics, Graduate School of Medicine, Osaka University, Suita, Osaka, Japan; 2 Topcon Advanced Biomedical Imaging Laboratory, Oakland, New Jersey, USA; 3 Department of Advanced Device Medicine, Graduate School of Medicine, Osaka University, Suita, Osaka, Japan; 4 Department of Innovative Visual Science, Graduate School of Medicine, Osaka University, Suita, Osaka, Japan; 5 Artificial Intelligence Center for Medical Research and Application, Osaka University Hospital, Suita, Osaka, Japan; 6 Department of Ophthalmology, Graduate School of Medicine, Osaka University, Suita, Osaka, Japan; 7 Structural Biophysics Group, School of Optometry and Vision Sciences, College of Biomedical and Life Sciences, Cardiff University, Cardiff, Wales, UK; 8 Integrated Frontier Research for Medical Science Division, Institute for Open and Transdisciplinary Research Initiatives, Osaka University, Suita, Osaka, Japan

**Keywords:** optical coherence tomography, deep-learning, 3D choroidal vasculature, choroidal pathology

## Abstract

**Purpose:**

The purpose of this study was to quantify choroidal vessels (CVs) in pathological eyes in three dimensions (3D) using optical coherence tomography (OCT) and a deep-learning analysis.

**Methods:**

A single-center retrospective study including 34 eyes of 34 patients (7 women and 27 men) with treatment-naïve central serous chorioretinopathy (CSC) and 33 eyes of 17 patients (7 women and 10 men) with Vogt-Koyanagi-Harada disease (VKH) or sympathetic ophthalmitis (SO) were imaged consecutively between October 2012 and May 2019 with a swept source OCT. Seventy-seven eyes of 39 age-matched volunteers (26 women and 13 men) with no sign of ocular pathology were imaged for comparison. Deep-learning-based image enhancement pipeline enabled CV segmentation and visualization in 3D, after which quantitative vessel volume maps were acquired to compare normal and diseased eyes and to track the clinical course of eyes in the disease group. Region-based vessel volumes and vessel indices were utilized for disease diagnosis.

**Results:**

OCT-based CV volume maps disclose regional CV changes in patients with CSC, VKH, or SO. Three metrics, (i) choroidal volume, (ii) CV volume, and (iii) CV index, exhibit high sensitivity and specificity in discriminating pathological choroids from healthy ones.

**Conclusions:**

The deep-learning analysis of OCT images described here provides a 3D visualization of the choroid, and allows quantification of features in the datasets to identify choroidal disease and distinguish between different diseases.

**Translational Relevance:**

This novel analysis can be applied retrospectively to existing OCT datasets, and it represents a significant advance toward the automated diagnosis of choroidal pathologies based on observations and quantifications of the vasculature.

## Introduction

The choroid plays an essential role in various physiological processes in the eyes and has been implicated in a range of pathologies. For example, age-related macular degeneration (AMD) is a leading cause of vision loss worldwide, which is associated with choroidal neovascularization in its advanced stages. In recent years, a condition called pachychoroid has attracted attention,^1^ whereby choroidal blood vessels dilate, which may damage the retinal pigment epithelium (RPE) and diminish retinal function. One of the pachychoroid related diseases is central serous chorioretinopathy (CSC), a non-inflammatory condition in which fluid accumulation between the retina and choroid leads to a retinal detachment and visual impairment.^1^ Accordingly, the noninvasive in situ visualization of the choroid, accompanied by a robust three dimensional (3D) quantification of choroidal vasculature, will be of fundamental importance for understanding and diagnosing a range of sight-threatening conditions and helping to develop new therapeutic strategies. The choroid has also been implicated in glaucoma and myopia,^2^^–^^6^ which are major causes of visual dysfunction affecting, respectively, over 80 million and 1.4 billion individuals worldwide.^7^^,^^8^ Previously, it was reported that patients with glaucoma had thicker choroid at the macular or peripapillary area than healthy subjects.^2^^,^^3^ For myopia, resent investigation suggests that choroidal thinning is only partially explained by axial elongation and that additional active mechanisms lead to choroidal thinning in myopia, such as alterations in blood vessels.^4^ Obtaining information about the status of choroid also has relevance for Vogt-Koyanagi-Harada disease (VKH) and sympathetic ophthalmia (SO), autoimmune, and inflammatory conditions with a similar pathogenesis that affect the choroidal vascular system.^9^ Recent reports have also indicated that imaging tissues deep in the eyes, such as the retina, choroid, and optic disc, can aid the diagnosis of nonocular pathologies, including autoimmune disease, hypertension, and diabetes.^10^

Choroidal tissue cannot be satisfactorily studied using conventional imaging methods, such as slit-lamp microscopy or the use of a fundus camera owing to the proximity of the RPE. Rather, indocyanine green angiography (ICGA) and optical coherence tomography (OCT) are the preferred techniques for evaluating the fine structure of the choroid and its vasculature.^11^^,^^12^ ICGA relies on the injection of dye to visualize blood flow in the choroid, followed by the collection of a series of time-lapse 2D images. As such, it lacks 3D information and also carries the risk of inadvertent leakage of the dye. Choroidal imaging by OCT has the advantage of being noninvasive, but the potential of imaging artifacts induced by the proximity of the retina may adversely affect the data. A recent work showed a 3D visualization of the choroidal vessels using OCT in comparison to ICGA.^13^ Here, we show how we can achieve 3D choroidal vessel volume visualization to interrogate the choroidal vasculature in a quantitative manner. Importantly, the technology can be applied to existing OCT datasets, offering widespread applicability.

## Methods

### Patients

This study was approved by the Osaka University Graduate School of Medicine Institutional Review Board (project # 09260-5, 17279-3). Patients with CSC, VKH, and SO were recruited by Osaka University Hospital from October 2012 to May 2019 adhering to the tenets of the Declaration of Helsinki. This is a retrospective case series study, thus, informed consent was not required. The study included 77 eyes of 39 healthy subjects (26 women and 13 men) who lacked any sign of retinal disease, optic disc malformation, or high myopia, 34 eyes of 34 patients (7 women and 27 men) with treatment-naïve CSC, and 33 eyes of 17 patients (7 women and 10 men) with VKH or SO.

### Diagnostic Criteria

A diagnosis of CSC adhered to published guidelines,^1^^,^^14^ based on a neurosensory detachment imaged by OCT in conjunction with the findings of fluorescein angiography (FA; i.e. leakage at the level of the RPE) and ICGA (hyperpermeability of choroidal vessels). Subjects were excluded if the CSC was accompanied by another eye disease, or if a severe RPE detachment caused significant OCT signal attenuation that resulted in an inability to get a signal from the choroid. Treatment was in accordance with standard therapeutic practice.^14^ A diagnosis of VKH or SO was made using diagnostic criteria based on data from slit-lamp examination, OCT, angiography (FA and ICGA), and lumbar puncture.^15^^,^^16^ Initial examinations were conducted 3 days after treatment because the severity of the retinal detachment, accompanied by vitreous opacification due to inflammation, prohibited observation of the choroid by OCT at the first consultation. Treatment was as described in previous reports.^15^^,^^17^^,^^18^

### Preprocessing of Volumetric OCT Data

**Figure 1.**
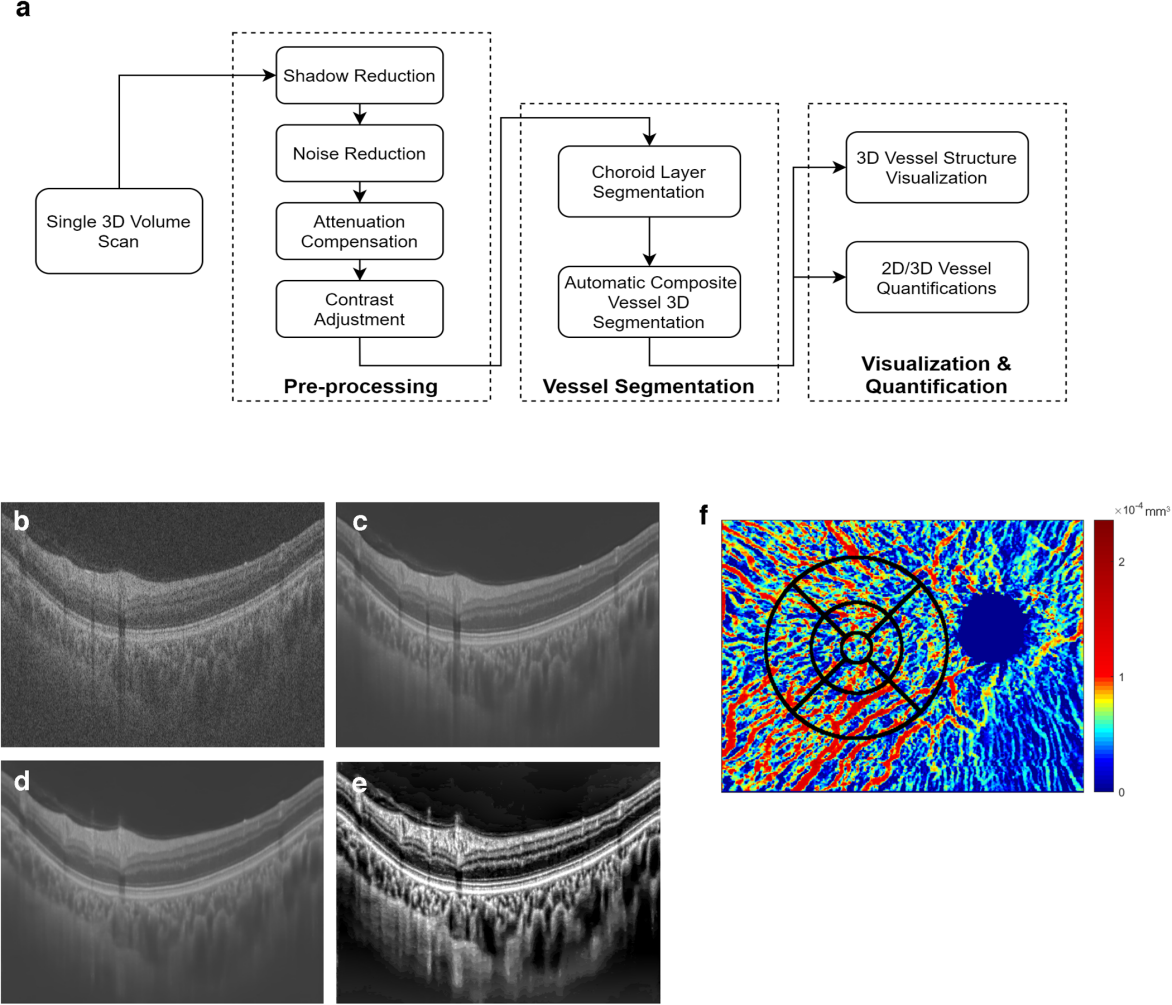
**Illustration of the processing protocol.** Data processing flowchart (**A**) accompanied by the original OCT image before preprocessing (**B**), after noise reduction (**C**), after shadow and noise reduction (**D**), and at the conclusion of the processing (**E**). The vessel volume map provides quantitative vessel volume information at each location and can be overlaid with an ETDRS grid to reveal focal changes to aid clinical decision making (**F**).

The OCT used was a swept-source instrument (SS-OCT; DRI-OCT; TOPCON, Tokyo, Japan), which acquired 12 mm × 9 mm B-scans over the macular area to provide 3D volume information. Data acquired from a single, routine OCT volumetric scan was used to generate the 3D vascular structure of the choroid for quantitative analysis. Figure 1a shows a flowchart of the approach. First, a series of preprocessing procedures are applied to the original volumetric OCT scan (Fig. 1b) to enhance the image quality and aid accurate choroidal vessel segmentation. A deep-learning (DL) based noise reduction method is then used to reduce the speckle noise in OCT images (Fig. 1c). The algorithm is trained and validated with all OCT scan patterns, including macular scans, disc scans, and wide scans, and can enhance the image quality to a level comparable to that of the 128× registration average.^19^ Third, a shadow reduction method is performed to minimize shadows cast by retinal vessels in the choroid (Fig. 1d) because these have a similar appearance to the choroidal vessels and if not removed will lead to artifacts during vessel segmentation.^19^ The algorithm minimizes the shadows by normalizing each A-line in the OCT volume with a filtered energy profile that compensates the reduced energy caused by shadowing; it is universally applicable to all scan patterns and does not change the contrast of the original image. Further, an attenuation compensation (Fig 1e) is executed to improve the image contrast in the deep choroid.^20^^,^^21^ Finally, a local contrast enhancement is used to improve vessel visualization and aid segmentation.^22^^,^^23^ Notably, our preprocessing method works on a single OCT volume obtained without any special data acquisition protocols, and thus offers a significant practical advantage in that it can be applied widely and retrospectively to existing databases to gain new insights into the vasculature of the choroid in health and disease. Recently, other techniques addressing speckle noise^24^^–^^27^ and shadow artifact^28^ have been proposed and can also be used as alternatives to our methods in the processing pipeline to achieve the quantitative analysis.

### Choroidal Vessel Segmentation and 3D Visualization

Vessel segmentation was conducted in the choroid, which is defined as the region between Bruch's membrane and the choroidal-scleral interface, using the TABS algorithm^29^ (Topcon DRI OCT Triton, version 10.16.003.02; Topcon, Tokyo, Japan). Segmentation results were manually inspected and corrections made if required. Within the choroid, vessels are segmented by a fully automated approach via a composite method that utilizes the contrast difference between the choroidal vessels and stroma.^30^^–^^33^ Here, we combine the results of local thresholding (Niblack) of the B-scan view,^30^^,^^34^ local thresholding of the C-scan view^32^ and global thresholding methods.^35^ B-scan view segmentation has been widely applied for choroidal vessel segmentation because of its relative simplicity,^30^^,^^34^^,^^35^ but often it does not work well with small vessels. The C-scan view images are generated by flattening the volume with respect to Bruch's membrane. Segmentation in a C-scan view serves to improve vessel connectivity, especially after shadow reduction, as C-scan images no longer contain confounding shadows from the retinal vessels. Moreover, because vessels at the same C-scan level have similar sizes, the segmentation works well for large and small vessels alike. On the other hand, the global thresholding method works best to segment the large dilated vessels in disease conditions when local thresholding methods struggle to perform. A composite segmentation result contains the detailed 3D choroidal vessel structure, whereas 3D stroma information is obtained from the inverse of the vessel segmentation. Our 3D segmentation is performed on a pixel-base basis, which offers a practical advantage in that both the 3D visualization and the quantification of the choroidal vessels can be achieved. The 3D segmentation is fed directly into rendering software^36^ to automatically create a 3D visualization of the choroidal vasculature. The clarity and detail achieved using the automated segmentation method described here shows that the technology outperforms previously reported automated procedures,^32^^,^^33^ and generates results that are comparable to those achieved by manual segmentation.^37^

The 3D segmentation also provides quantification of the choroidal vessels. To achieve this, pixel resolution is calculated from the scan parameters embedded in each scan, and the volume is calculated by multiplying the number of pixels by the pixel resolution. Measurements of total choroidal volume and choroidal vessel volume, moreover, can be combined with the choroidal stroma volume map calculated at each A-line to create a choroidal vessel volume map, which can be applied widely or aggregated in a specified region. The potential distortion caused by tilting of the slab is not corrected for in the present work. The vessel volume index is calculated as the ratio between the vessel volume and the total choroidal volume. Figure 1f illustrates how an overlay of a choroidal vessel volume map with an early treatment diabetic retinopathy study (ETDRS) grid can be applied to derive regional quantifications and reveal focal changes in vascular pathology.

### Statistical Analysis

Statistical comparisons between each disease group for vessel volume, choroidal volume, and vessel index were performed by a Kruskal-Wallis test with Dunn's multiple comparisons test. Statistical comparison of the clinical time course between each disease group with respect to the vessel volume, choroidal volume, and vessel index were performed by a 1-way ANOVA test with Dunnett multiple comparison test. A *P* value of < 0.05 was considered statistically significant. The performance of the vessel volume, choroidal volume, and vessel index and their cutoff points were assessed using the receiver operator curve (ROC) space. The area under the curve (AUC) is commonly used to evaluate the classification models. All analyses were performed using Prism 9 software, version 9.0.0 (Graph Pad Software, San Diego, CA, USA).

## Results

### Quantitative Visualization of Choroidal Vessel Structure

**Figure 2.**
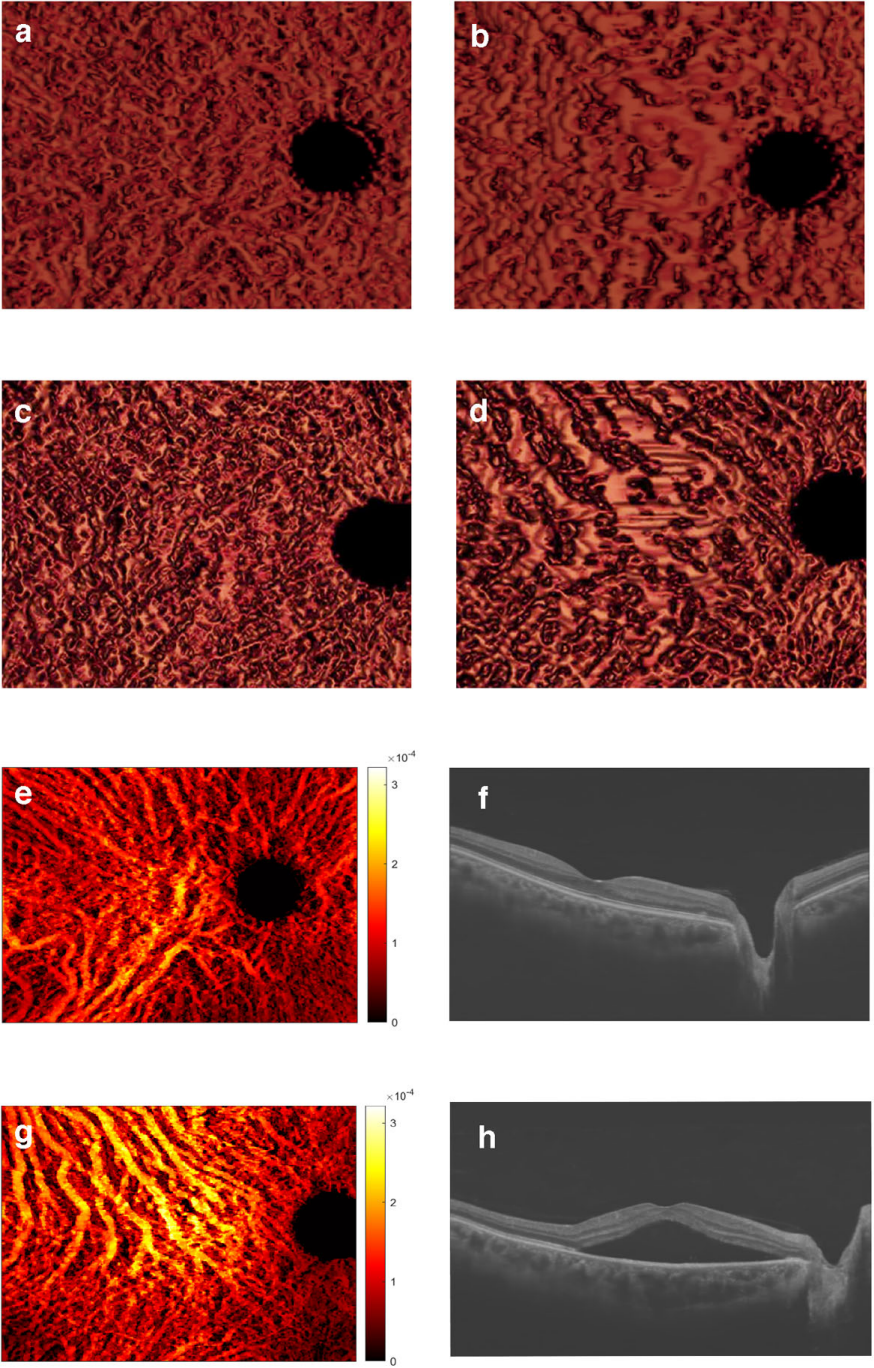
**Visualization of choroidal vessel structure by deep-learning OCT.** Choroidal vessel structure in a healthy subject (**A, B, E, F**) and a patient with CSC patient (**C, D, G, H**). Vessel volume maps (**E, G**) are accompanied by the noise-reduced and shadow-compensated OCT B-scans (**F, H**). All vessel volume maps are quantitative, and the color bars have the same scale in units of mm^3^. It can be observed directly from the color of the vessel volume map that the patient with CSC has a larger choroidal vessel volume. The 3D choroidal vasculature can be observed from the retinal side (**A, C**) and from the sclera side (**B, D**). The patient with CSC (**C, D**) presents with an increased vessel thickness and a distorted vessel shape compared to the normal subject (**A, B**). Clear vessel structures are revealed in both cases despite the existence of significant amounts of subretinal fluid and a thick choroid in the diseased condition.

The 3D visualization of the vascular structure of the choroid described here allows for depth-dependent observations that are not achievable using existing modalities. This can uncover pathological changes in choroidal vasculature, as illustrated by the visualization of a normal eye (Figs. 2a, 2b) and that of a CSC eye (Figs. 2c, 2d). At the retinal side (see Figs. 2a, 2c) the thickness of the blood vessels is the same in both eyes, but at the sclera side (see Figs. 2b, 2d) vessels are manifestly thicker and more distorted in the CSC eye. The vessel volume maps generated from the 3D vessel segmentation uniquely exhibit spatially colocalized structures and contain quantitative volume information at each location en face. As such, it provides significantly enhanced quantitative information for a deeper appreciation of the choroidal vasculature and the pathogenesis of choroid-related vision loss. To illustrate this, vessel volume maps are shown in Figure 2 for healthy (Fig. 2e) and CSC eyes (Fig. 2g), accompanied by the noise-reduced and shadow-compensated OCT B-scans across the fovea (Figs. 2f, 2h). The vessel volume map clearly reveals vessel structures, not only in the healthy eye but also in the diseased CSC eye despite the existence of a relatively large amount of subretinal fluid in the CSC eye with its thick choroid. The intensity values in the vessel volume map correspond to the total vessel volume at each location, and the color bars (units = mm^3^) are depicted using the same scale in both eyes. This color/intensity display enables an appreciation of a larger vessel volume and more dilated vessels in CSC eyes when compared to normal eyes (Fig. 2g). Supplemental video files show the normal 3D rotation and CSC 3D rotation video.

### Comparison With Indocyanine Green Angiography 

**Figure 3.**
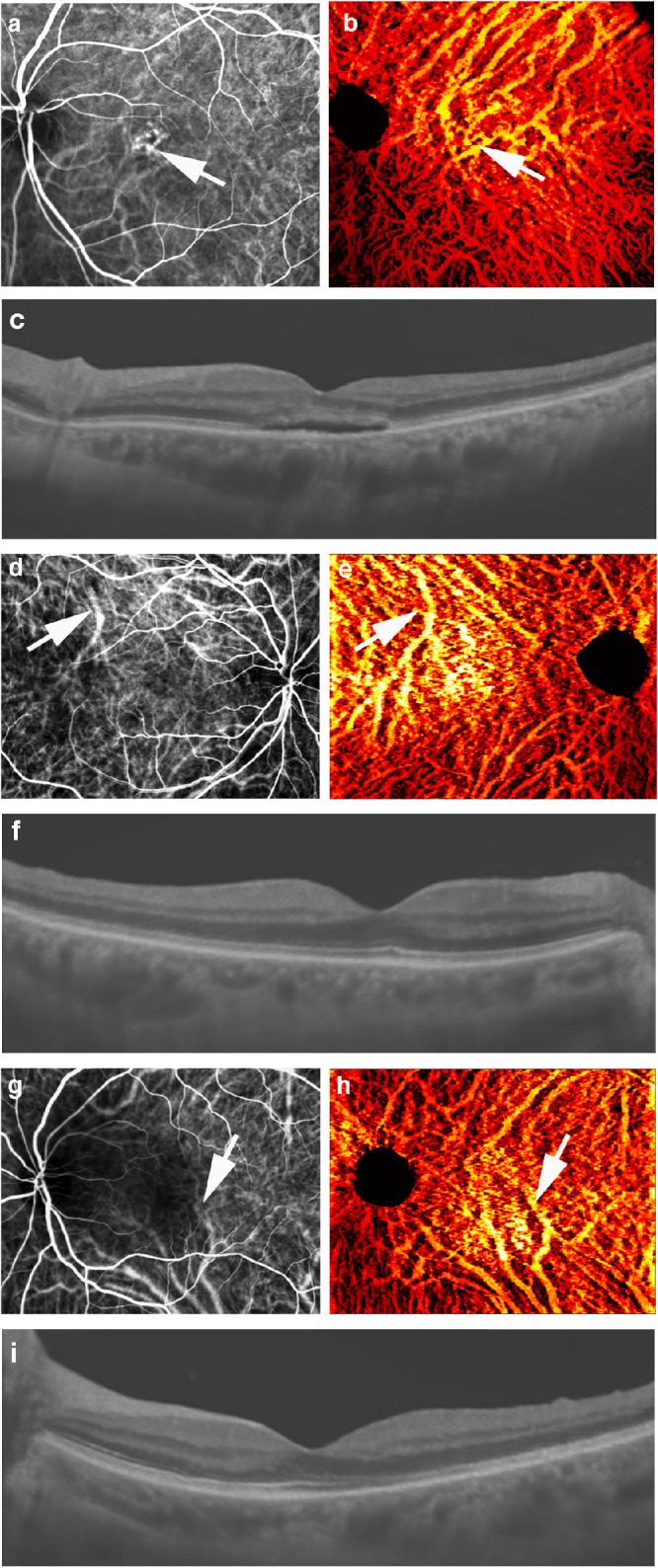
**Choroidal vessel volume maps compared to ICGA images.** Visualization of choroidal vessel structure in one eye of a patient with CSC (**A–C**) and both eyes of a patient with VKH (**D–I**) presented as ICGA images (**A, D, G**), vessel volume maps (**B, E, H**), and noise-reduced and shadow-compensated OCT B-scans (**C, F, I**). The dark areas in ICGA panels indicate subretinal fluid and the malformed choroidal vessels underneath. Because the malformed choroidal vessels are usually dilated and leaky, their structures and anatomical changes are difficult to observe on ICGA (**A, D, G**), and are more clearly observed in vessel volume maps (**B, E, H**). In areas where choroidal vessels are identified by ICGA, vessel structures in vessel volume maps match well (*white arrows*).

Figure 3 presents vessel volume maps (Figs. 3b, 3e, 3h) and B-scans (Figs. 3c, 3f, 3i) alongside ICGA images (see Figs. 3a, 3d, 3g) of one eye of a patient with CSC (see Figs. 3a, 3b, 3c) and both eyes of an individual with VKH (right eye: see Figs. 3d, 3e, 3f; left eye: Figs. 3g, 3h, 3i). In areas where choroidal vessels are clearly imaged by ICGA, the vessel structure in the OCT vessel volume map matches well to that of the ICGA image, including identification of high-volume malformed vessels (arrows). In other areas, choroidal vessels are more readily observed in the vessel volume map than in the ICGA image. Thus, in most cases when the choroid is observable by ICGA, the vessel volume map represents a comparable, or even superior, imaging modality.

### Clinical Course of Choroidal Vessel Changes After Treatment

**Figure 4.**
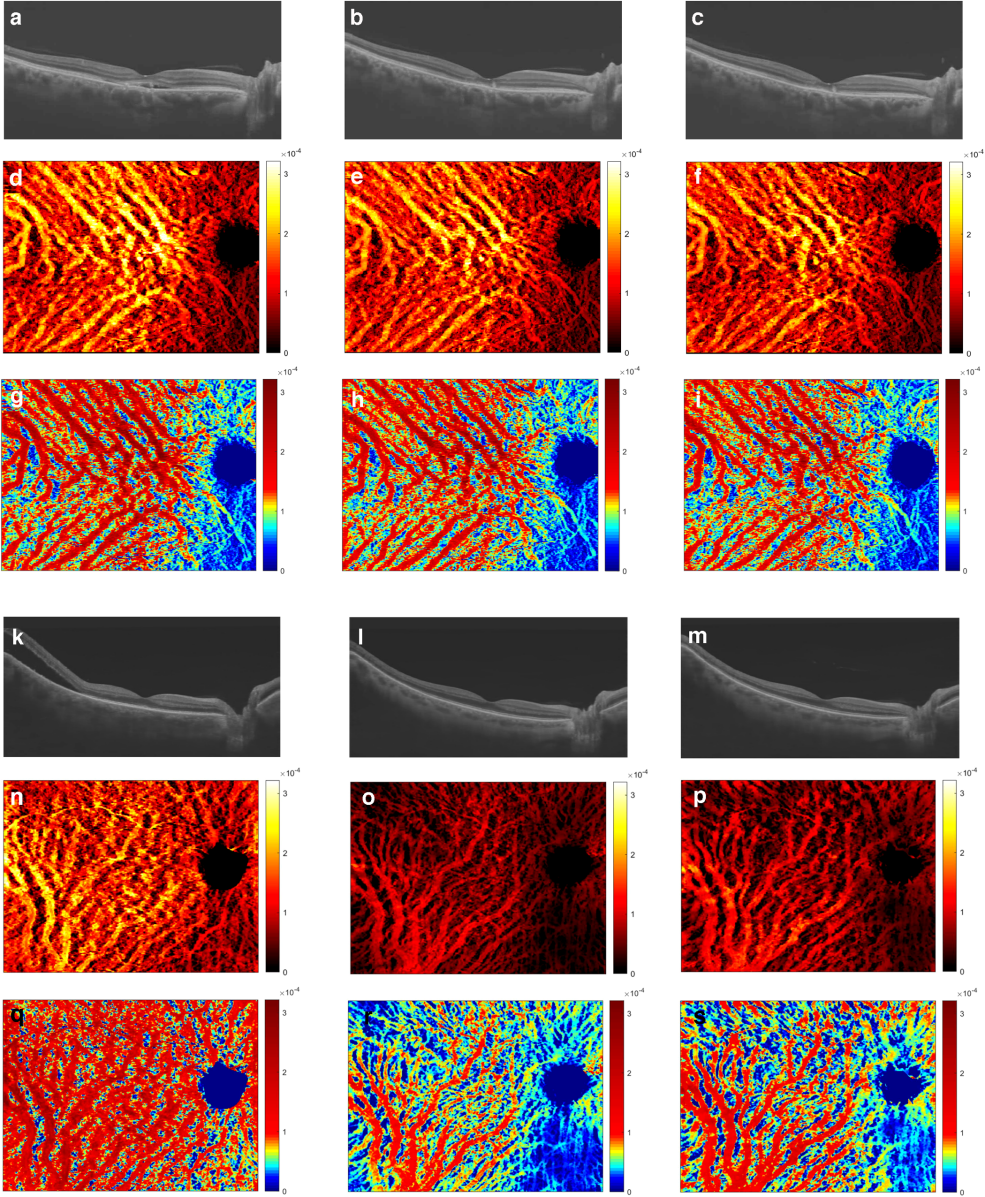
**Clinical course of CSC and VKH following treatment.** Processed OCT B-scans for a patient with CSC (**A–C**) and a patient with VKH (**K–M**), accompanied by warm (**D–F, N–P**) and cool (**G–I, Q–S**) vessel volume colormaps. The warm colormap generally highlights the vasculature better, whereas the cool colormap tends to be more suited for the inspection of vessel volume change. Scans were taken immediately after treatment (**A, D, G**) and at 1 month (**B, E, H**) and 3 months (**C, F, I**) post-treatment for CSC, and at 3 days (**K, N, Q**), 1 month (**L, O, R**), and 6 months (**M, P, S**) after treatment for VKH. Images are not appreciably altered for the patient with CSC, but a treatment effect is evident on inspection of colourmaps for the patient with VKH. This observation is confirmed by statistical analysis.

**Figure 5.**
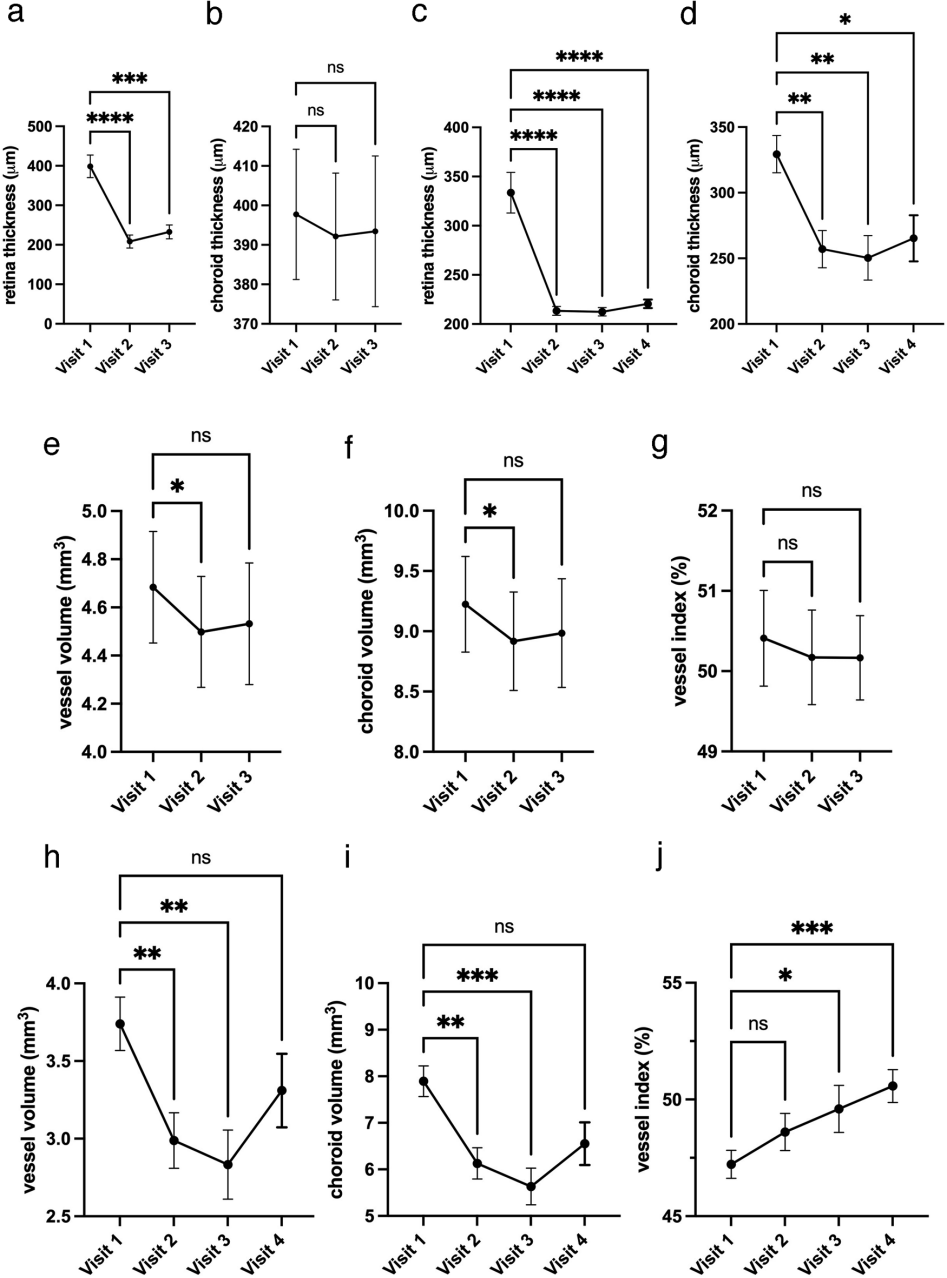
Statistical analysis of retinal thickness, choroidal thickness, choroidal volume, vessel volume, and vessel index changes in patients with CSC and patients with VKH following treatment. Retinal and choroidal thicknesses monitored across the clinical course in CSC (**A, B**) and VKH (**C, D**) show significant changes in all metrics except choroidal thickness in CSC **B**. Choroidal volume, vessel volume, and vessel index were measured in the central 6 mm-diameter areas of images for the CSC group (23 eyes of 23 patients) and the VKH group (34 eyes of 17 patients). The CSC group shows a significant change in choroidal volume, vessel volume at 1 month (visit 2), but not at 3 months (visit 3) (**E, F**). Vessel index post treatment in the patient with CSC shows no significant change during clinical course (**G**). The VKH group shows a significant change in vessel volume and choroidal volume at 1 month and 3 months (visits 2 and 3) (**H, I**). The vessel index post-treatment in VKH shows significant change during the clinical course at 3 and 6 months (visits 3 and 4) (**J**). *P < 0.05, **P < 0.01, ***P < 0.001, ****P < 0.00001. Statistical analysis confirms the trend.

As well as its value as a diagnostic tool, the OCT-based vessel volume map technology can be used to monitor disease progression and ascertain the effect of treatment. This is exemplified in Figure 4, which shows the clinical course in one eye of a patient with CSC (see Figs. 4a–i) and the clinical course in one eye of a patient with VKH (see Fig. 4k–t) in the 3 months following treatment, as revealed by the vessel volume maps. The patients are different to those described in Figure 3, and the treatments involved photocoagulation for CSC and corticosteroid administration for VKH. Corresponding preprocessed OCT data are also shown (see Figs. 4a–c, 4k–m), and indicate how an assessment of choroidal thickness measured by OCT B-scans and vessel volume maps correlates. Our analysis indicates that a warm colormap tends to highlight the vasculature better, whereas a cool colormap is generally more suited for the inspection of volume change. The findings underscore how the response to treatment can vary, with a fairly dramatic vessel volume change observed for the patient with VKH, but only a slight change seen in the treated eye of the patient with CSC. We further tested the capacity of the technology to gauge post-treatment choroidal changes by measuring choroidal metrics in 34 CSC eyes and 33 VKH eyes. Traditional 2D analyses of the retinal and choroidal thicknesses were also carried out (Figs. 5a, 5b, 5c, 5d). The analysis indicated that the retinal thickness decreases dramatically after treatment in both CSC and VKH, whereas the choroidal volume only changed in the VKH case. A minimal change in vessel volume was seen after CSC treatment (see Figs. 5a, 5b), measuring 4.68 ± 0.23 mm^3^ initially, 4.50 ± 0.23 mm^3^ at 1 month, and 4.53 ± 0.25 mm^3^ at 3 months. Likewise, changes in choroidal volume, at 9.22 ± 0.40 mm^3^ initially, 8.92 ± 0.41 mm^3^ at 1 month, and 8.07 ± 0.45 mm^3^ at 3 months, were not high. However, a statistically significant reduction was apparent after VKH treatment (3.74 ± 0.17 mm^3^ initially, 2.99 ± 0.18 mm^3^ at 1 month, 2.38 ± 3.29 mm^3^ at 3 months, and 2.83 ± 0.24 mm^3^ at 6 months; see Figs. 5e, 5f, 5h, 5i). Color maps and quantitative analysis revealed that in the initial stages of treatment both CSC and VKH/SO patient groups were possessed of large choroid vessel volumes. Subsequently, the patients with VKH/SO showed a more dramatic reduction in vessel volume 1 month after treatment (see Figs. 4q, 5h), indicating a better response to treatment than for patients with CSC. During the post-treatment clinical course, the vessel index (i.e. the ratio between vessel volume and choroidal volume) increased and reached a normal condition 3 to 6 months following treatment in the patients with VKH/SO group, whereas no significant improvement was found in patients with CSC (see Figs. 5g, 5j). This indicates that the parenchymal volume decreases at a higher rate than the blood vessel volume, and suggests that treatment in our patient cohort suppresses parenchymal inflammation in VKH/SO.

### Diagnostic Value of Choroidal Volume and Vessel Volume Measurements

**Figure 6.**
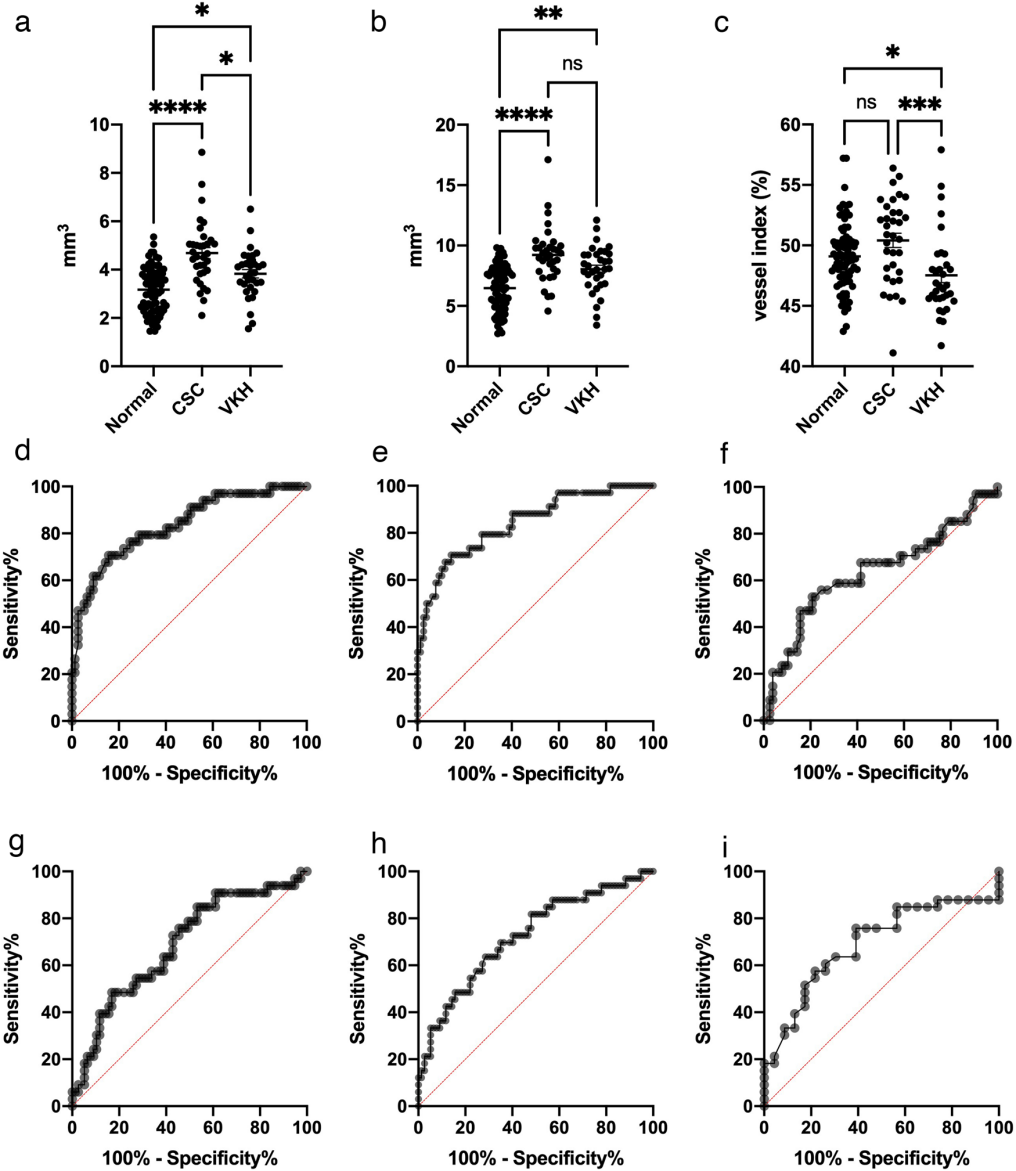
Quantitative analysis of choroidal vessel volume, choroidal volume and vessel index for the diagnosis of CSC and VKH. Vessel volume (*P* < 0.0001) and choroidal volume (*P* < 0.0001) in CSC and VKH eyes (**A, B**) are significantly different to normal. Vessel index in VKH/SO eyes is significantly different from normal, but is not significant for CSC (**C**). The area under the curve (AUC) values from the vessel volume, choroidal volume and vessel index, respectively (**D–I**), are 0.84 and 0.84 for CSC (**D, E**), and 0.69 and 0.72 for VKH (**G, H**). The AUC for the vessel index is significant in the VKH/SO group (AUC = 0.69) but not in CSC (**F, I**). To diagnose CSC, the criteria is a vessel volume greater than 4.14 mm^3^ (sensitivity 70.59% and specificity 84.42%) or a choroidal volume greater than 8.47 mm^3^ (sensitivity 69.57% and specificity 85.71%). For VKH, a vessel volume greater than 3.57 mm^3^ (sensitivity 63.64% and specificity 61.04%) or a choroidal volume greater than 7.55 mm^3^ (sensitivity 69.707% and specificity 64.94%) are diagnostic criteria. To distinguish VKH from normal, the vessel index is less than 48.10% (sensitivity 72.73% and specificity 64.94), and to distinguish VKH from CSC, the vessel index is less than 48.05% (sensitivity 72.73% and specificity 73.53%). *P < 0.05, **P < 0.01, ***P < 0.001, ****P < 0.00001. Statistical analysis of data acquired from within the 6 mm-diameter inner region of the ETDRS grid confirms the trend.

To further investigate if the vessel volume and choroidal volume can become useful diagnostic indicators of choroidal disease, we compared metrics from CSC and VKH small-group series with age-matched control data from healthy eyes (Fig. 6). This uncovered statistically meaningful differences for both CSC and VKH pathologies when compared with healthy eyes. The average vessel volume and average choroidal volume in the healthy group, measured in the central 6-mm diameter circle of the ETDRS chart (see Fig. 1f), are 3.17 ± 0.11 mm^3^ and 6.47 ± 0.21 mm^3^, respectively. For comparison, both vessel volume and choroidal volume exhibited higher values in the CSC and VKH groups. Specifically, for the CSC group, the average vessel volume and average choroidal volume were measured to be 4.68 ± 0.23 mm^3^ and 9.23 ± 0.40 mm^3^, significantly higher than the normal values of 3.83 ± 0.17 mm^3^ and 8.04 ± 0.34 mm^3^, respectively (*P* < 0.0001 for both metrics). Similarly, the average vessel and choroidal volumes for the VKH/SO group were also significantly higher at 3.83 ± 0.17 mm^3^ and 8.04 ± 0.34 mm^3^, respectively (vessel volume: *P* < 0.01, choroidal volume: *P* < 0.001; see Figs. 6a, 6b). The vessel index in VKH/SO eyes, at 47.52 ± 0.59, was less than in normal eyes at 49.09 ± 0.32, and when CSC and VKH eyes were compared, it transpired that the vessel index was significantly lower in VKH eyes, at 47.52 ± 0.59, than in CSC eyes at 50.41 ± 0.59 (versus normal: *P* < 0.05, versus CSC: *P* < 0.001; see Fig. 6c).

The ROC curves to classify CSC and VKH as measured from vessel and choroidal volumes are shown in Figure 6 for CSC (see Figs. 6d, 6e) and VKH (see Figs. 6g, 6h). AUC values measured from the vessel volume and choroidal volume, respectively, are 0.84 and 0.84 for CSC (see Figs. 6d, 6e), and 0.69 and 0.72 for VKH (see Figs. 6g, 6h). The AUC value measured from the vessel index is only significantly different in the VKH/SO group (AUC = 0.69; see Figs. 6f, 6i). In CSC, the vessel volume is greater than 4.14 mm^3^ (sensitivity 70.59% and specificity 84.42%), whereas the choroidal volume is greater than 8.47 mm^3^ (sensitivity 69.57% and specificity 85.71%). In VKH, the vessel volume is greater than 3.57 mm^3^ (sensitivity 63.64% and specificity 61.04%), whereas the choroidal volume is greater than 7.55 mm^3^ (sensitivity 69.707% and specificity 64.94%). Moreover, for VKH compared with healthy eyes, the vessel index is less than 48.10% (sensitivity 72.73% and specificity 64.94%; see Fig. 6i), whereas VKH compared with CSC eyes revealed that the vessel index was less than 48.05% (sensitivity 72.73% and specificity 73.53%).

## Discussion

To the best of our knowledge, this represents the first quantitative 3D volume analysis of choroid metrics in healthy and diseased eyes, with previously quantitative reports of choroidal vascularity performed in 2D without the visualization of vasculature,^31^^,^^34^ or limited to healthy subjects and 2D or 3D visualization.^13^^,^^35^^,^^38^^,^^39^ It is notable that the new analytical technology described here allows the clinician to seek to pinpoint focal changes in the choroidal vasculature because the vessel volume maps can be formatted to scrutinize small, selected quadrants. The combination of visual observation and quantitative analysis, noninvasively and at defined locations, will be highly valuable to diagnose, monitor, and assess the response to treatment for a wide range of ocular diseases with choroidal involvement. This may satisfy the long-sought yet unmet need of the clinician to interrogate the choroidal vasculature when choroid-related disease is suspected, and to do this in a noninvasive manner.

**Figure 7.**
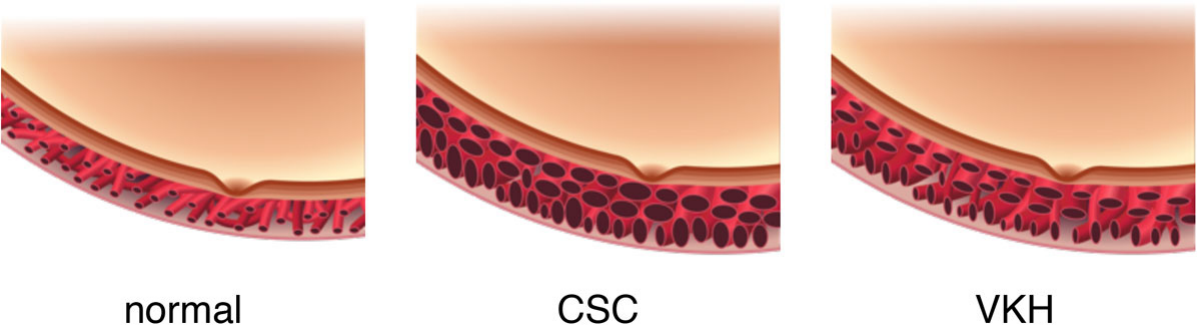
**The pathophysiology for CSC and VKH/SO.** Although both CSC and VKH/SO eyes exhibit a higher vessel volume and a higher choroidal volume than normal, the vessel index (i.e. the ratio between the vessel and choroidal volumes) are different in the two diseases. Specifically, the vessel index in CSC eyes is similar to healthy eyes, whereas the vessel index in VKH/SO eyes is less than it is in healthy or CSC eyes.

We have shown that the quantification of choroidal vasculature via an OCT processing pipeline is effective in diagnosing, distinguishing, and monitoring disease progression in two chosen choroid-related pathologies, CSC and VKH/SO. Patients with VKH/SO display a larger choroidal volume, as do patients with CSC. However, the vessel index in VKH is less than the value found in both healthy and CSC eyes. A pathophysiological consideration of this suggests that patients with VKH/SO have a larger choroidal volume outside the choroid vessel than patients with CSC do. Figure 7 illustrates the different characteristics of choroidal vessels in healthy patients, and patients with CSC and VKH/SO. We note that the analysis of choroidal vessels in the present work considers choroidal vessels from Sattler's layer to Haller's layer without dividing them into sublayers, so the illustration is based on an average of the choroidal vessel size. In typical VKH, it is possible to diagnose the pathological condition from the retinal and choroidal morphology in the acute phase in 2D OCT images. However, when the images show a morphology similar to CSC (e.g. subretinal fluid or thick choroid) our 3D vessel analysis is needed to distinguish VKH from CSC by use of the vessel index metric in conjunction with other clinical symptoms at the initial clinical time point. Although the eyes of patients with CSC have larger choroidal volumes and choroidal vessel volumes compared with the eyes of unaffected subjects, we did uncover a subset of “healthy” eyes (13 eyes: greater than 4.14 mm^3^) that also display relatively large choroidal volumes and choroidal vessel volumes. We speculate that these eyes may be at risk of developing CSC, and we will closely monitor this group of individuals using the new metrics obtained from the vessel volume maps as biomarkers for CSC as part of a preventative medicine strategy.

A current limitation of the approach presented here is the need for a manual inspection to achieve layer segmentation in highly diseased cases. Nevertheless, research is ongoing and developments toward a fully automated segmentation are on the horizon, which will dispense with the need for manual input, even when disease is present. One potential caveat, however, is that excessive turbidity of the intraocular fluid or substantive bleeding within the eye would adversely affect the optical penetration of OCT. We are aware that due to limited light penetration into edematous tissues, OCT signal intensity from choroidal vessels may be insufficient to analyze severe pathologic change in the presence of large volumes of subretinal fluid. A strength of the analysis described lies in the fact that it can be conducted on existing OCT datasets, and thus represents significant added value facilitating widespread retrospective adoption of the technology on existing databases to gain new insights into the pathobiology of choroidal disease. The visualization and quantifications, moreover, can be applied to clinical investigations of choroid-related pathologies other than CSC and VKH/SO, and these include AMD (where the efficacy of treatments such as anti-VEGF injections could be tracked) and pathological myopia, where the value of various therapeutic interventions could be evaluated.

## Supplementary Material

Supplement 1

Supplement 2
